# Greenhouse gas emissions convergence in Spain: evidence from the club clustering approach

**DOI:** 10.1007/s11356-020-08214-4

**Published:** 2020-07-05

**Authors:** Nicholas Apergis, Antonio J. Garzón

**Affiliations:** 1 School of Business, Law and Social Sciences, University of Derby, Derby, UK; 2 Faculty of Economics and Business Sciences, University of Seville, Seville, Spain

**Keywords:** Greenhouse gas emissions, Convergence, Club clustering, Spain

## Abstract

This study examines the convergence of greenhouse gas emissions per capita across the 19 Spanish regions using the Phillips-Sul club convergence approach over the period spanning from 1990 to 2017. The results indicate the presence of four clubs which converge to different equilibria in emissions per capita and three clubs in terms of income per capita, which involves different regions. These findings suggest that mitigation policies should explicitly consider the presence of different clubs of regions with different convergence paths in terms of emissions and income per capita and address the distributional effect of transfers across regions.

## Introduction

Greenhouse gases emissions, their impact on climate change and global warming have become one of the main concerns of policymakers over the recent decades, leading countries to the signing of international agreements, such as the Kyoto Protocol, aimed at reducing emissions on the global level. According to such agreements, countries are committed to reduce their emissions primarily through national level measures. This fact implies that they are primarily responsible in designing their own mitigation policies in order to meet specific targets.

An important issue for policymakers at national level is the distribution of emissions across regions, as well as their evolution over time. A key question is whether differences in emissions across regions tend to increase or decrease over time (Burnett 2016). This issue could affect the design of mitigation policies and the principles used to share the burden of emission reduction across regions. If emissions converge over time, while their overall growth rates decrease, the distributional impact of the mitigation schemes, such as equal per capita allowances, is less concerning for policymakers (Apergis et al. 2017), given that transfers across regions are reduced as they converge to the same level of emissions per capita. However, if differences in emissions tend to increase over time, the mitigation policies could have distributional costs across regions, resulting in increasing transfers of resources or the reallocation of emission-intensive industries. Therefore, policymakers should take explicitly into account such regional differences in the designing of mitigation policies (Burnett 2016; Apergis and Payne 2017), including other indicators beyond the uniform per capita allowances in the emission allocation schemes, such as the ability to pay or adjustment costs. This issue has attracted the interest of researchers, who have studied the presence of convergence in emissions at sub-national level across the US states (Aldy 2007; Bulte et al. 2007; Li et al. 2014; Payne et al. 2014; Apergis and Payne 2017; Apergis et al. 2017), as well as across Chinese provinces (Huang and Meng 2013; Wang and Zhang 2014; Zhao et al. 2015; Wu et al. 2016; Yang et al. 2016), using different methodologies and definitions of convergence.

In this paper, we extend the research on greenhouse emissions convergence to the case of Spanish regions. Spain, as a member of the European Union, is framed in the Horizon 2030 programme, which established a target of reducing by 40% greenhouse emissions by 2030 from their 1990 levels (European Commission 2014). Since 1990, Spanish emissions have evolved in the same direction along with economic growth, following an increasing trend until 2007, where a turning point was achieved and emissions started to decrease. Nevertheless, emissions have relatively stabilized at 2013, despite higher economic growth rates experienced over the last years (Ministerio para la Transición Ecológica 2019). In 2017, emission levels were 17.9% above 1990 levels, the benchmark for reduction targets, implying that an important effort should be made to achieve its commitment. If we focus on the emission intensity, measured as emissions per GDP unit, the picture is different. In particular, a downward trend can be observed since 1995, with an increasing pace after 2005 (Ministerio para la Transición Ecológica 2019), implying that emission growth has been lower than GDP growth during the same period.

In regard to the regional economic performance, several works suggest that the convergence process of Spanish regions stopped after the 1980s (Leonida and Montolio 2004; Tortosa-Ausina et al. 2005; Castro 2007; Diez-Minguela et al. 2018) and it has given rise to the formation of two convergence clubs or groups of regions (Montolio and Leonida; Tortosa-Ausina et al. 2005; Montañés and Olmos 2014; Diez-Minguela et al. 2018). Given that income growth and greenhouse gas emissions have been linked at the national level, it is important to test whether this fact holds for Spanish regions; convergence patterns would imply that regional emissions are diverting or converging over time, arising important policy implications in relation to the design of mitigation measures and their economic impact. If different convergence patterns are found, a uniform mitigation scheme would involve sizeable and increasing transfers of income across regions, favouring the introduction of other principles for sharing the burden of emission reduction. Therefore, the goal of this paper is to explore the convergence patterns for greenhouse gas emissions across all Spanish regions. Our study extends the literature on the long-run convergence in regional emissions per capita on certain fronts. First, as the first study on Spanish emissions convergence, we focus our attention on all 19 regions geographically dispersed across Spain (Andalusia, Aragon, Asturias, Balearic Islands, Basque Country, Canary Islands, Cantabria, Castille-La Mancha, Castille-Leon, Catalonia, Extremadura, Galicia, Madrid, Murcia, Navarre, Rioja, Valencia, Ceuta and Melilla). Second, while previous studies in emissions convergence have primarily implemented unit root and cointegration tests, which rely on the assumption of variable stationarity, this current work employs the time-varying nonlinear approach recommended by Phillips and Sul (2007). This approach identifies that convergence does not depend on the assumptions regarding the stationarity of variables and allows us to test both overall convergence and the identification of convergence clubs across regions.

## Methodology

The panel includes *N* = 19 Spanish regions, while the number of time frequency is *T* = 28. The approach employs a time-varying common factor *y*_*it*_ defined as:

1 $$ {y}_{it}={\delta}_{it}{\mu}_t $$

*y*_*it*_ is the log of emissions (income) per region *i* at time *t*, while *μ*_*t*_ is a common trend component, and *δ*_*it*_ is a time-varying idiosyncratic component that captures time as well as individual specific effects; it indicates the distance between *y*_*it*_, and the common factor, *μ*_*t*_, is the common stochastic trend, while *δ*_*it*_ yields:

2 $$ {\delta}_{it}={\delta}_i+{\sigma}_i{\upxi}_{it}L{(t)}^{-1}{t}^{-\upalpha} $$

*δ*_*i*_ is fixed and ξ_*it*_~iid(0, 1) across regional factor *i* = 1, 2, …, *N* and weakly dependent over time *t*; *σ*_*i*_ is an idiosyncratic scale parameter; *L*(*t*) is a varying function of time, with *L*(*t*) → ∞ and *t* → ∞. 1The null hypothesis of convergence is *H*_O_ : *δ*_*i*_ = *δ* and *α*_i_ ≥ 0 against the alternative hypothesis: *H*_A_ : {*δ*_*i*_ = *δ* for all *i* with *α*_*i*_ < 0 } or {*δ*_*i*_ ≠ *δ* for some *i* with *α*_*i*_ ≥ 0, or α_i_ < 0}.

*y*_*it*_ and *μ*_*t*_ do not need to be trend stationary. Phillips and Sul (2007) employ the quadratic distance measure, *H*_*t*_, as follows:

3 $$ {H}_t={N}^{-1}\sum \limits_{i=1}^N{\left({h}_{it}-1\right)}^2 $$

*h*_*it*_ is the relative transition coefficient,

4 $$ {h}_{it}=\frac{\mathrm{c}{\mathrm{p}}_{\mathrm{it}}}{N^{-1}\sum \limits_{i=1}^N{\mathrm{c}\mathrm{p}}_{it}}=\frac{\delta_{it}}{N^{-1}\sum \limits_{i=1}^N{\delta}_{it}} $$

This coefficient captures the transition path with respect to the panel average. When there is a common behaviour across individual regions, *h*_*it*_ = h_t_ across *i*, then convergence occurs when *h*_*it*_ → 1 for all *i* as *t* → ∞; however, in the case that convergence does not hold, the distance remains positive as t →  ∞ .Following Phillips and Sul (2007), *L*(*t*) = log*t* in the decay model (2), so the empirical logt regression can be used to test for convergence clubs as follows:

5 $$ \log \left(\frac{H_1}{H_t}\right)-2\log \left(\log t\right)=a+\upgamma \mathrm{logt}+{\upvarepsilon}_{\mathrm{t}} $$

for *t* = rT, rT + 1, …, *T* where *r* > 0 set on the interval [0.2, 0.3]. For *γ* = 2a, the null hypothesis is a one-sided test of $\hat{\gamma}\ge 0$, against $\hat{\gamma}<0$. To avoid estimates that are potentially weakly time-dependent, the least squares estimate of *γ* is based on heteroskedasticity and autocorrelation consistent standard errors.^2^

### Data

**Fig. 1:**
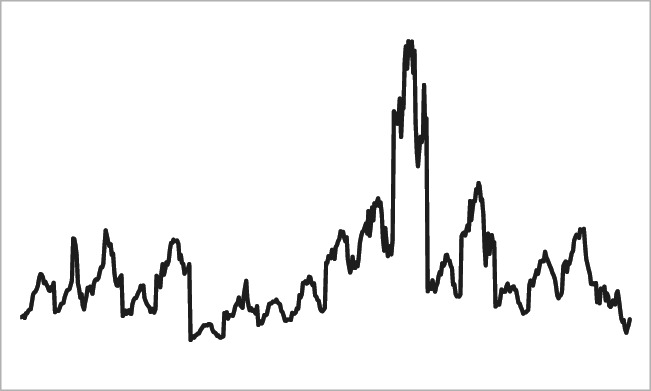
Carbon emissions across all Spanish regions

The dataset spans from 1990 to 2017 on an annual frequency basis and covers the 17 Spanish Autonomous Communities (Andalusia, Aragon, Asturias, Balearic Islands, Basque Country, Canary Islands, Cantabria, Castille-La Mancha, Castille-Leon, Catalonia, Extremadura, Galicia, Madrid, Murcia, Navarre, Rioja and Valencia) and the two Autonomous Cities of Ceuta and Melilla. Greenhouse gas emissions data by regions are obtained from the Spanish Informative Inventory Report elaborated by the Spanish National Inventory System. Greenhouse gas emissions per capita are measured in tonnes of carbon dioxide equivalents (tCO_2_eq) and include the emissions of CO_2_, CH_4,_ N_2_O, SF_6_, HFC and PFC. These data are defined as emissions divided by total population. Data on population are obtained from the Spanish Regional Accounts database published by the Spanish Statistical Office (INE). Real GDP data by region are sourced from de la Fuente (2019) and are expressed in thousands of constant 2010 euros, while GDP per capita is measured by dividing real GDP by population. Table 1 offers some descriptive statistics, while Fig. 1 illustrates the panel picture of carbon emissions across all Spanish regions.

**Table 1:** Descriptive statistics

Variable	Mean	SD	Max	Min
Emissions per capita	0.0099	0.0053	0.0329	0.0030
Real GDP per Capita	4.319	4.679	4.523	4.006

*SD* standard deviation

### Empirical analysis

Panel A of Table 2 reports the panel convergence results for *r* = 0.30.^3^ The first row displays the results testing for convergence in emissions per capita across all regions. The coefficient estimate is *γ*= − 0.588 with a *t*-statistic, ${t}_{\hat{\gamma\ }}$= − 4.5116, and statistically significant at 1%, indicating the rejection of the null hypothesis of overall panel convergence. Next, the analysis proceeds to determine whether we can identify club clusters. The results from the club clustering algorithm illustrate the presence of three distinct clubs. Club 1 consists of the regions of Extremadura, Aragon and Asturias, with *γ*= 0.0935 and ${t}_{\hat{\gamma}}=$ 0.4028, which fails to reject the null hypothesis of convergence. Club 2 encompasses the regions of Rioja, Navarre, Cantabria, Galicia and Castille-Leon, with *γ* = 0.0939 and ${t}_{\hat{\gamma}}=0.2160$ which again fails to reject the null hypothesis of convergence. Club 3 contains the regions of Andalusia, Murcia, Valencia, Canary Islands, Basque Country, Catalonia, Balearic Islands and Castille-La Mancha, with *γ* = 0.0705 and ${t}_{\hat{\gamma}}=0.3324$, failing again to reject the null hypothesis of convergence. Finally, Club 4 includes the regions of Madrid, Ceuta and Melilla, with *γ* = 0.7993 and ${t}_{\hat{\gamma}}=1.2051$, failing to reject the null hypothesis. Note that Phillips and Sul (2007, 2009) suggest that using the sign criterion may lead to over-estimation of the true number of clubs. To address this potential issue, the analysis also performs club-merging tests via regression (3) to determine whether merging adjacent numbered clubs into larger clubs is relevant. Panel B of Table 2 shows that tests of merging clubs support the merger of the Club 1 and Club 2, which implies that these regions are converging at a slower pace to the same level.

**Table 2:** Tests of club convergence (emissions per capita)

Panel A: Club convergence		
Full Sample (19 regions): Madrid, Basque Country, Navarre, Catalonia, Aragon, Rioja, Balearic Islands, Castille-Leon, Asturias, Cantabria, Galicia, Murcia, Valencia, Andalusia, Canary Islands, Castille-La Mancha, Extremadura, Ceuta, Melilla		
$\hat{\gamma\ }$= −0.5880***		
${t}_{\hat{\gamma}}$ = −4.5116		
Club 1 (3 regions): Extremadura, Aragon, Asturias		
$\hat{\gamma\ }$= 0.0935		
${t}_{\hat{\gamma}}$= 0.4028		
Club 2 (5 regions): Rioja, Navarre, Cantabria, Galicia, Castille-Leon		
$\hat{\gamma\ }$= 0.0939		
${t}_{\hat{\gamma}}$= 0.2160		
Club 3 (8 regions): Andalusia, Murcia, Valencia, Canary Islands, Basque Country, Catalonia, Balearic Islands, Castille-La Mancha		
$\kern1em \hat{\gamma\ }$= 0.0705		
${t}_{\hat{\gamma}}$= 0.3324		
Club 4 (3 regions/cities): Madrid, Ceuta and Melilla		
$\hat{\gamma\ }$= 0.7993		
${t}_{\hat{\gamma}}$= 1.2051		
Panel B: Merging clubs		
Clubs	$\hat{\gamma\ }$	${t}_{\hat{\gamma}}$
Club 1 + Club 2	− 0.071	− 0.28
Club 2 + Club 3	− 0.461	− 3.54***
Club 3 + Club 4	− 0.379	− 2.63***

*Notes*: Testing for the one-sided null hypothesis $\hat{\gamma}\ge 0$ against $\hat{\gamma}<0$, the analysis makes use of the critical value *t*_0.05_ =  − 1.65156 across all cases

***p ≤ 0.01

The findings reported in Panel A in Table 3 illustrate the presence of three clubs of convergence on income per capita basis. The test for full sample rejects the null hypothesis of convergence, with $\hat{\gamma\ }$= − 0.5280 and ${\mathrm{t}}_{\hat{\upgamma}}$ = − 14.2760. The first club of convergence consists of the regions of Madrid and Basque Country, which are the regions with the highest income per capita, i.e. a higher converging GDP per capita level. A second club is formed by the regions of Extremadura, Rioja, Navarre, Cantabria, Murcia, Valencia, Galicia, Aragon, Asturias, Castille-Leon, Catalonia and Balearic Islands that includes regions with high income per capita converging to a lower GDP per capita level (i.e. Catalonia), and regions with low and middle income per capita, with an increasing trend and converging to a higher GDP per capita (i.e. Extremadura). Finally, the third club is made up of the regions of Andalusia, Canary Islands, Castille-La Mancha, Ceuta and Melilla, highlighting low income regions which are converging to a lower level of GDP per capita than Clubs 1 and 2.

**Table 3:** Tests of club convergence (GDP per capita)

Panel A: Club convergence		
Full Sample (19 regions): Madrid, Basque Country, Navarre, Catalonia, Aragon, Rioja, Balearic Islands, Castille-Leon, Asturias, Cantabria, Galicia, Murcia, Valencia, Andalusia, Canary Islands, Castille-La Mancha, Extremadura, Ceuta, Melilla		
$\hat{\upgamma\ }$ = − 0.5280***		
${\mathrm{t}}_{\hat{\upgamma}}$ = − 14.2760		
Club 1 (2 regions): Madrid, Basque Country		
$\hat{\upgamma\ }$ = 1.8889		
${\mathrm{t}}_{\hat{\upgamma}}$ = 7.9258		
Club 2 (12 regions): Extremadura, Rioja, Navarre, Cantabria, Murcia, Valencia, Galicia, Aragon, Asturias, Castille-Leon, Catalonia, Balearic Islands		
$\hat{\upgamma\ }$= − 0.0321		
${\mathrm{t}}_{\hat{\upgamma}}$= − 0.8784		
Club 3 (5 regions/cities): Andalusia, Canary Islands, Castille-La Mancha, Ceuta and Melilla		
$\hat{\upgamma\ }$ = 1.1624		
${\mathrm{t}}_{\hat{\upgamma}}$ = 6.3106		
Panel B: Merging clubs		
Clubs	$\hat{\upgamma\ }$	${\mathrm{t}}_{\hat{\upgamma}}$
Club 1 + Club 2	−0.366	−10.06***
Club 2 + Club 3	−0.242	−6.50***

*Notes*: Testing for the one-sided null hypothesis $\hat{\gamma}\ge 0$ against $\hat{\gamma}<0$, the analysis makes use of the critical value *t*_0.05_ =  − 1.65156 across all cases

***p ≤ 0.01

## Conclusion and policy implications

This study tested overall emissions convergence across Spanish regions. To this end, the analysis used the club-clustering approach by Phillips and Sul (2007). The findings documented the presence of different convergence patterns in terms of emissions per capita and income per capita. Some of the high-income regions, such as Madrid, Catalonia or Basque Country, are converging to lower levels of emissions per capita than some low- or middle-income regions, such as Extremadura, Aragon or Asturias. This implies that a regional mitigation scheme based only in per capita allowances would entail significant transfers of income from high emission-intensive regions to low emission-intensive regions, some of the latter being the wealthiest regions, thus producing a regressive redistribution between regions and increasing the differences in income levels. This evidence recommends policies towards reducing emissions which also take account of the ability to pay, that is, emissions allowances should be allocated not only based on the size of population (equal per capita emissions), but also inversely to GDP per capita, reducing the differences between the Spanish regions in terms of income.

These findings recommend that the level of economic activity, measured by GDP per capita, is not the main determinant of the emissions per capita level across Spanish regions. Other aspects, such as the regional economic structure, could also play an important role in determining emissions levels, which suggest the need of further research in emissions convergence across Spanish industries and the policy implications that could entail.

## Footnotes

1 The parameter α is the rate at which the cross-section variation over the transitions decays over time to zero.

2 −2 log(log*t*) acts as a penalty function. The omission of this term under the alternative hypothesis would have an upward bias on the least square estimator of *γ*. 

3 Phillips and Sul (2007) suggest that *r* = 0.3 is a satisfactory selection in terms of both size and power.
